# Plant-Waste-Derived Sorbents for Nitazoxanide Adsorption

**DOI:** 10.3390/molecules28155919

**Published:** 2023-08-07

**Authors:** Artur Sokołowski, Katarzyna Jędruchniewicz, Rafał Kobyłecki, Robert Zarzycki, Krzysztof Różyło, Haitao Wang, Bożena Czech

**Affiliations:** 1 Department of Radiochemistry and Environmental Chemistry, Faculty of Chemistry, Maria Curie-Sklodowska University in Lublin, 20-031 Lublin, Poland; asokolowski98@gmail.com (A.S.); katarzyna.jedruchniewicz@mail.umcs.pl (K.J.); 2 Department of Advanced Energy Technologies, Częstochowa University of Technology, Dąbrowskiego 73, 42-201 Częstochowa, Poland; rafal.kobylecki@pcz.pl (R.K.); robert.zarzycki@pcz.pl (R.Z.); 3 Department of Herbology and Plant Cultivation Techniques, University of Life Sciences in Lublin, 20-033 Lublin, Poland; krzysztof.rozylo@up.lublin.pl; 4 Tianjin Key Laboratory of Environmental Technology for Complex Trans-Media Pollution, Key Laboratory of Pollution Processes and Environmental Criteria (Ministry of Education), College of Environmental Science and Engineering, Nankai University, Tianjin 300350, China; envwang@nankai.edu.cn

**Keywords:** adsorption, waste-derived adsorbents, biochar, nitazoxanide

## Abstract

The increased application of drugs during the COVID-19 pandemic has resulted in their increased concentration in wastewater. Conventional wastewater treatment plants do not remove such pollutants effectively. Adsorption is a cheap, effective, and environmentally friendly method that can accomplish this. On the other hand, maintaining organic waste is required. Thus, in this study, plant waste-derived pelletized biochar obtained from different feedstock and pyrolyzed at 600 °C was applied for the adsorption of nitazoxanide, an antiparasitic drug used for the treatment of SARS-CoV-2. The adsorption was fast and enables one to remove the drug in one hour. The highest adsorption capacity was noted for biochar obtained from biogas production (14 mg/g). The process of NTZ adsorption was governed by chemisorption (k_2_ = 0.2371 g/mg min). The presence of inorganic ions had a detrimental effect on adsorption (Cl^−^, NO_3_^−^ in 20–30%) and carbonates were the most effective in hindering the process (60%). The environmentally relevant concentration of DOM (10 mg/L) did not affect the process. The model studies were supported by the results with a real wastewater effluent (15% reduction). Depending on the applied feedstock, various models described nitazoxanide adsorption onto tested biochars. In summary, the application of carbonaceous adsorbents in the pelletized form is effective in nitazoxanide adsorption.

## 1. Introduction

Nitazoxanide (2-acetyloxy-*N*-5-nitro-2-thiazolyl benzamide, NTZ) is an antiparasitic drug that also shows a broad spectrum of antiviral activity [1]. In the early 2000s, nitazoxanide was approved for the therapy of diarrhea caused by *Cryptosporidium* species and *G. intestinalis* in children and adults. [2]. Among the other tested antiparasitic drugs thiabendazole, albendazole, and ivermectin are listed [3,4]. Nitazoxanide is also effective against anaerobic bacteria, protozoa, trematodes, cestodes, nematodes, and even viruses [5,6]. Due to the antiviral properties of nitazoxanide, in 2020 when the COVID-19 pandemic started, NTZ was used to treat patients infected with SARS-CoV-2 [2,7]. In a patient’s body, nitazoxanide is hydrolyzed into tizoxanide (desacetyl-nitazoxanide) (half-life 7.3 h) and one-third of the oral dose of nitazoxanide is excreted with urine and the rest with feces [8] resulting in the presence in wastewater [9]. Although the concentrations of antiparasitic drugs in wastewater or the environment are relatively low (Table 1), their poor removal in wastewater treatment plants has been observed. This is evidence that novel effective methods for its removal are required.

Among others, adsorption seems to be a promising removal method [11,12,13] due to its low cost, short time, and the possibility of using different wastes for different products in order to meet the requirements of the circular economy. Additionally, the transformation of waste towards precious products satisfies Sustainable Development Goals. The solution for problematic organic wastes is their transformation towards biochar [14]. The unquestionable advantage of biochar is the possibility of application of various feedstocks (straw [15], wood [16], food wastes [14], sewage sludge [17], animal manures [18], etc.). Additionally, a wide range of applied temperatures (350–750 °C) or gases (steam, CO_2_, N_2,_ etc.) [19] during pyrolysis or per-and post-modification of biochar [20] makes it the best solution for environmental application. Final product properties such as surface area, pore size, hydrophobicity, and pH are dependent on feedstock and conditions of the pyrolysis process [20,21,22].

The use of waste materials as feedstock for the synthesis of biochar has many benefits. One of them is the low price of biochar. Another advantage is that the waste materials are processed safely. It makes less waste go to landfills, which reduces methane emission and reduces the microbiological risk [22,23,24]. Biochar may have a big specific surface area and high adsorption capacity. Additionally, they have less negative impact on the environment compared to other adsorbents. All of this makes them great adsorbents which may be successfully used in wastewater treatment [20]. Biochar is used for the removal of various substances such as phenols, polynuclear aromatics, pesticides, pharmaceuticals, dyes, and inorganics from water [10,20]. The problem in studies with biochar is its powdered form that requires additional separation after the process [25]. The solution will be pelletized feedstock [26], thus wheat straw pellets, sunflower husk pellets, and pelletized residues from biogas production were used for the preparation of the biochar. The dense structure of the adsorbent is superior in practical application and enabled easier removal after the process.

The aim of the presented studies was the comparison of the effectiveness of various waste-derived pelletized biochar in the adsorption of NTZ from water. The capacity of NTZ removal was established for all adsorbents, using both kinetic studies and isotherm modeling. Testing kinetics enables to establish the rate-limiting step and the sorption mechanism starting from the mass transfer of adsorbate, its diffusion, and finally the surface reaction on the adsorbent [27]. Kinetics was tested using four well-known models: pseudo-first-order (PFO), pseudo-second-order (PSO), Elovich model, and Intraparticle Diffusion model (IPD). In the Lagergren PFO model, the adsorption rate with time is directly proportional to the difference in saturation concentration thus adsorption occurs through diffusion through the interface. In PSO the rate-limiting step is chemical sorption or chemisorption. In this condition, the adsorption rate is dependent on adsorption capacity not on the concentration of adsorbate [27]. Elovich’s model describes the activated chemical adsorption onto heterogeneous surfaces [28]. The intraparticle Diffusion model assumes the adsorbate diffusion in the pores inside as the mass transfer step [29]. The sorption capacity can be estimated using isotherm modeling [30] and four well-known models: Langmuir (L), Freundlich (F), Temkin (T), and Dubinin-Radushkevich (DR) were examined. L model describes a monolayer filling of non-interacting molecules to homogeneous binding sites with equivalent sorption energies [31], whereas the F model describes the process onto heterogeneous surfaces [32]. In the T model a uniform distribution of bounding energy up to some maximum bonding energy is assumed, whereas DR describes the impact of adsorbent porosity as well as defines the character of sorption (physical or chemical) depending on the value of adsorption energy [33].

The objectives of the presented studies were: (i) the determination of the applicability of waste-derived pelletized adsorbents in the pollutants removal from water matrix; (ii) the estimation of the effect of the parameters governing the adsorption process onto biochar surface (organic and inorganic substances); (iii) efficiency of the NTZ removal from different water and wastewater matrix. All tested conditions were used to estimate the possibility of the practical application of waste-derived biochar.

## 2. Results and Discussion

### 2.1. Biochar Physicochemical Properties

**Figure 1:**
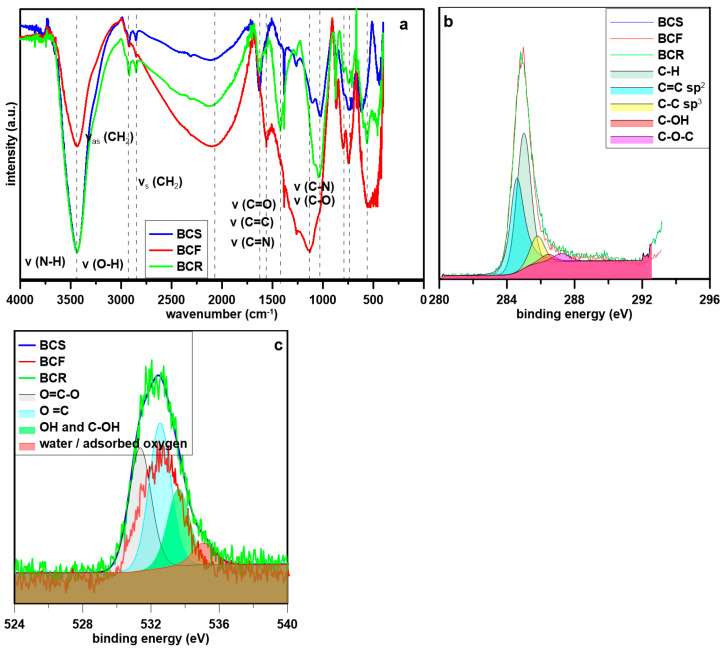
The spectroscopic analysis of tested materials: (**a**) FTIR spectra, (**b**) XPS C peak, and (**c**) XPS O peak.

The obtained material differed in physicochemical properties (Table 2, Figure 1a–c). In general, BC was characterized by low surface area but the application of residues from biogas production for BC was efficient and enabled to obtain material with the highest surface area (26.05 m^2^/g). BCR possessed also the highest micropore volume with simultaneously the lowest pore diameter (10.68 nm). The mostly broad pores were noted in BCS (95.41 nm). Porosity is a key parameter in adsorption where pore-filling or sieving effects are considered [12]. The highest ash content e.g., presence of inorganics was noted in BCR, which contained also K (4.05 wt%), Ca (1.35 wt%), Mg (0.76 wt%), Fe (0.38 wt%), S (0.24 wt%), Si (0.14 wt%), Cl (0.14 wt%), and P (0.09 wt%) that was evidenced from SED-EDS mapping.

The highest amount of carbon (C%) was noted in BCF (Table 2), the material with the lowest hydrophilicity (O/C ratio) and polarity ((O + N/C) ratio). On the other hand, the mostly hydrophilic/polar surface was noted in BCR, which contained the highest amount of O%. These results may indicate that BCS and BCF may be recommended for the adsorption of rather hydrophobic pollutants, whereas BCR is for hydrophobic compounds [34].

The FT-IR studies revealed that the surface of BC was characterized by the presence of various surface functional groups (Figure 1a). At all curves, the presence of peaks confirming the presence of O-H and/or N-H stretching vibrations (about 3445 cm^−1^) was noted. The double peaks at 2940 cm^−1^ and 2840 cm^−1^ corresponded to the asymmetric and symmetric stretching of the aliphatic CH_2_ group. The other peaks implied that lignin (peak at about 1620 cm^−1^—aromatic C=C or C=O stretching vibration or 1430 cm^−1^ C–H deformation) was present in all BC but with different intensities. The presence of a band at 1620 cm^−1^ may be connected with the presence of amino groups (C=N stretching) and/or amides (N-H bending) [35]. Peak noted at 2090 cm^−1^ indicated the presence of conjugated C=C and C≡C bonds with the highest content in BCF and the lowest in BCS was noted indicating higher graphitization of BCF in comparison to BCR and BCS. Amides were also present that confirmed the peaks at 1620 cm^−1^ (C=O) and 1550 cm^−1^ (C–N) [36]. BC contained also inorganic carbonates (stretching of –C=O at 1430 cm^−1^ and bending –C=O inorganic carbonates at 870 cm^−1^ in BCR [37], which was consistent with data presented in Table 2. The peak at 1020 cm^−1^ was indicating for SiO_2_ presence in BCS and BCR. Additionally, polycyclic aromatic hydrocarbons (PAHs) were noted in BC which was implied by the presence of the three-peak group at 874 to 750 cm^−1^ (aromatic C-H bands) [38].

The carbon peaks of all BC looked similar in the XPS spectrum (Figure 1b) and the presence of different carbon structures was confirmed. In General, XPS analysis revealed that carbon is present mostly in the form of C-H bonds (peak at 285 eV, 45.5 at% for BCS, 50.7 at% for BCF), and interestingly in minority in BCR (38.7 at%). The other form of carbon, C=C sp^2^ (peak at 284.57 eV) [39], constituted a slightly lower percentage (35.5% and 37.6%, for BCS and BCF respectively), whereas in BCR was predominant (41.8%). The presence of other peaks for C–C sp^3^ [40] (peak at 285.79 eV), C–OH (286.43 eV), C–O–C (peak at 287.22 eV), C=O [40] (288.10 eV), –COO-289.06 eV) and –COOR (290.18 eV) [41] indicated lower impact of these functionalities (below 10 at%). Oxygen in all BC was present, whereas the intensity of the O peaks in the XPS spectrum (Figure 1c) was not similar, with the lowest revealed by BCF. Oxygen was present in the form of carboxyl (peak at 531.3 eV) (25.9 at% in BCS, 27.3 at% in BCF, 33.8 at% in BCR) [40], carbonyl (peak at 532.6 eV) (34.5 at% in BCS, 38.9 at% in BCF, 39 at% in BCR), and hydroxyl groups (peak at 533.6 eV) (21.2 at% in BCR, 28.4 at% in BCF, 32.5% in BCS) [42]. Also, adsorbed water or oxygen was determined (peak at 534.9 eV) (below 10 at% in all BC) [40]. 

**Figure 2:**
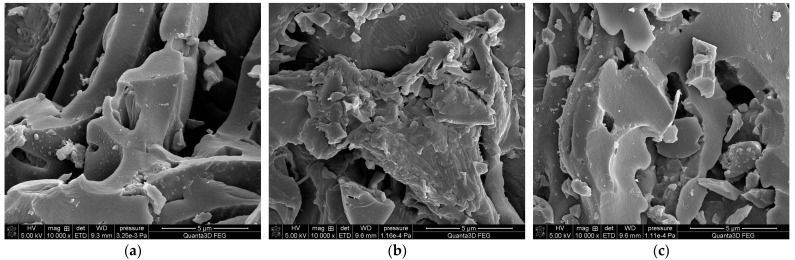
SEM images of tested materials: (**a**) BCS, (**b**) BCF, and (**c**) BCR.

The morphology of the tested materials was examined with SEM (Figure 2). The structure of BC is generally densely “packed” (Figure 2a,b) but some pores were observed. However, SEM images clearly confirmed the presence of voids and channels in the structure of BC [43]. The diameter of the pores was not uniform and dependent on the applied feedstock. In BCR the diameter of pores was lower than 1 µm (BCR Figure 2c). In turn, it can be seen that the morphology of BCS was slightly different from the other materials, and the layered structure was observed. Whereas the morphology of BCF was rather showing plane/non-porous surface. The surface of BCS and BCR seems to be rougher. These observations are consistent with data obtained from porosity (Table 2). Small bright fragments noted in the BCF SEM image are confirming the presence of inorganic constituents [35]. 

### 2.2. Adsorption Studies

**Figure 3:**
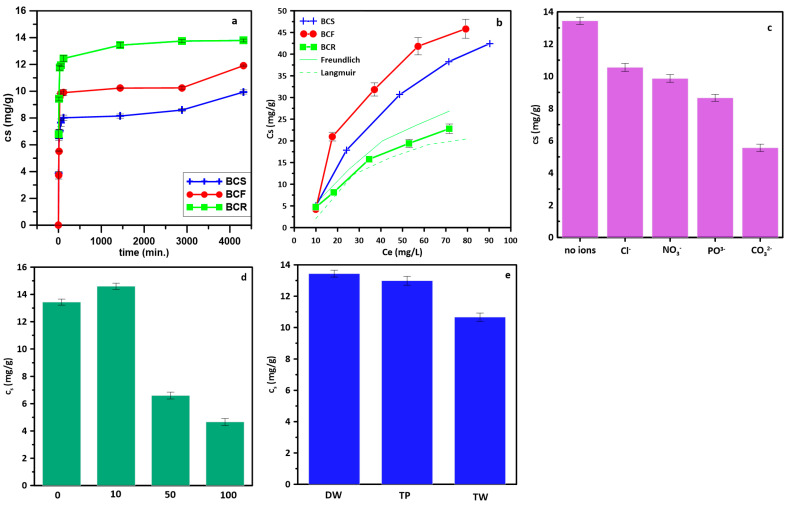
Adsorption studies of NTZ onto tested BC: (**a**) kinetics c_NTZ_ = 20 mg/L m_BC_ = 100 mg; (**b**) C_NTZ_ = 10–100 mg/L, m_BC_ = 100 mg; (**c**) effect of inorganic ions c_ion_ = 0.005 M; (**d**) effect of dissolved organic matter, c_TA_ = 10–100 mg/L; (**e**) effect of water matrix: DW—distilled water, TP—tap water, TW—treated wastewater.

The highest amount of adsorbed NTZ was observed on BCR (Figure 3a) almost 14 mg/g. In general, the process of sorption was rather fast and the sorption equilibrium was maintained for one hour indicating potential practical application. In general, the maximum adsorption capacity of all samples increased in the following order BCS < BCF < BCR.

The adsorption of ionic compounds is affected by their ionization state. Generally, the value of pKa of NTZ was 10.62 (chemdrug.com) indicating low acidic properties of the drug and that in the water environment (neutral pH) NTZ will be not ionized. The surface of the BC is negatively charged (ζ potential about −40 mV: BCS—−43.93 mV, BCF—−42.58 mV, and BCR—−35.7 mV). Thus the electrostatic interactions are not the main driving force for interactions of BC and NTZ [44]. 

The process of NTZ adsorption followed mainly PSO kinetics (Table 3, R^2^ > 0.99) indicating that the adsorption rate was combined with the number of binding sites and governed by chemisorption. The low fitting to the other kinetic models excludes physisorption and intraparticle diffusion as rate-limiting processes in tested adsorption. PSO was also noted for adsorption onto different carbon-based materials such as GAC [45] and biochar [46]. To establish the mechanism of adsorption, isotherm modeling was employed (Figure 3b, Table 4). What is interesting, the process followed different regimes depending on the applied feedstock. For BCS, the Temkin model revealed the highest fitting, whereas Dubinin-Radushkevich for BCF and Langmuir for BCR. Temkin model is describing the heterogeneity of the energy of the active sites on the adsorbent. Temkin’s equation assumes that the adsorption heat of molecules in the layer decreases linearly, which may be due to adsorbent-adsorbate interactions, and adsorption is characterized by an even distribution of binding energy [47], which was the highest in the case of BCS, 144.53 kJ/mol. In the Dubinin-Radushkevich model, the distribution of pores in adsorbents to follow Gaussian energy distribution is assumed [48], and the highest energy was noted for BCF, 253.27 kJ/mol. In the Langmuir model, a monolayer coverage is assumed [49] and the highest sorption capacity was noted for BCR, 418.55 mg/g. 

The process of sorption is dependent on the presence of co-solutes that may compete with adsorbate with active sites. In the presence of inorganic ions (Figure 3c), the efficiency of the process was lower. Common inorganic ions such as chlorides and nitrates revealed the lowest detrimental effect and about 20–30% lowered adsorption was noted. The highest reduction in NTZ adsorption (almost 60%) was noted in the presence of carbonates. 

The presence of DOM in the natural environment may impact the adsorption [18]. At environmentally relevant concentrations (up to 10 mg/L) DOM acts as the factor increasing the adsorption of NTZ (Figure 3d). DOM may chelate the substances in the environment thus preventing the adsorption or may be sorbed onto an adsorbent surface introducing new sites and increasing the adsorption [50]. At lower concentrations, the adsorption was increased, whereas at higher concentrations the competition with active sites on the adsorbent surface. The results from the studies on the effect of inorganic ions and dissolved organic matter were used for predicting the efficiency of NTZ removal from the water matrix. Tap water did not affect NTZ adsorption significantly (Figure 3e), whereas in treated wastewater the adsorption was at about 20% lower, suggesting that dissolved organic matter had higher impact on NTZ adsorption onto tested biochar.

Generally, adsorption results from the interaction between different pores: both micropores and mesopores [44]. The process of the adsorption of NTZ onto tested samples was governed by different interactions. For the description of adsorption onto carbonaceous materials, several mechanisms are described including electrostatic, hydrophobic interactions, and hydrogen bond (including charge-assisted hydrogen bond) formation [46,51]. Hydrogen bonding between carbonyl groups (COO^−^) and hydroxyl oxygen or protonated amine could also be responsible for NTZ adsorption. In summary, the application of carbonaceous adsorbents is effective and carbonaceous adsorbents can be easily modified to improve antiparasitic drug adsorption.

It is difficult to compare the obtained sorption results with literature data as nitazoxanide is not tested sorbate. Thus, the comparison may be performed only considering the antiparasitic activity of drugs and carbonaceous materials (Table 5). Interestingly, not the extent of surface area was the key parameter governing the adsorption, as materials with the highest surface area were not revealing the highest sorption capacity. The results obtained by us fit into this where the Langmuir sorption capacity of BCR was very high (almost 420 mg/g), but the surface area was low, 26 m^2^/g.

## 3. Materials and Methods

### 3.1. Biochar Production and Characterization

Biochar (BC) was produced by pyrolysis of pelletized biomass feedstock in a pilot reactor at 600 °C in the N_2_ atmosphere for 3 h. Due to the applied material, wheat straw-derived biochar was labeled as BCS, sunflower husk-derived BC- as BCF, and biochar obtained from residues from biogas production was labeled BCR. The obtained BC was milled and sieved and a fraction < 2.0 mm was used for adsorption studies. The porosity of the tested biochar (S_BET_ surface area, micropore volume, and pore diameter) was analyzed in ASAP 2420 Analyzer (Micromeritics, Norcross, GA, USA), and the elemental analysis (content of C, H, N) was performed in CHN/CHNS EuroEA3000 Elemental Analyzer (EuroVector, Pavia, Italy). Surface characteristics, e.g., the presence of the surface functional groups was analyzed by spectroscopic methods. FTIR spectra were collected in Bio-Rad Excalibur 3000 MX spectrometer equipped with photoacoustic detector MTEC300, and XPS (survey, O, C, N peaks) were examined in UHV (Prevac, Rogów, Poland). Ash content was calculated after the exposure of 1 g of biochar to 760 °C for 6 h in the muffle furnace (MagmaTherm, Kraków, Poland). Total carbon (TC), inorganic carbon (IC), and total organic carbon (TOC) of biochar were estimated in the Shimadzu TOC/N analyzer. The zeta potential measurements were carried out in the pH value range of 3–10 (Zetasizer 3000, Malvern Instruments with 0.1 M HCl or 0.1 M NaOH used for pH correction. The surface morphology was examined by Scanning Electron Microscopy equipped with EDS (Quanta 3D FEG, FEI). 

### 3.2. Adsorption Studies 

Nitazoxanide and tannic acid were obtained from Sigma-Aldrich (Poznań, Poland), whereas NaCl, NaNO_3_, Na_2_CO_3,_ and Na_3_PO_4_ were purchased in POCH (Poland). The ultrapure water was produced by a Millipore Direct Q 3UV water purification system (Millipore, Livingston, UK). Adsorption was performed using the batch technique in 50 mL Falcon tubes, where 100 mg of biochar was contacted with 50 mL of a solution containing 20 mg/L NTZ in distilled water without modification of pH (pH of the solution was 6.4). For isotherm modeling concentrations 10–100 mg/L were applied. The effect of inorganic ions: Cl^−^, PO_4_^3−^, NO_3_^−^, CO_3_^2−^ was tested using respective sodium salts at the concentration of 0.005 M. As the representative of the dissolved organic matter (DOM), a tannic acid solution was chosen. The effect of the water matrix was established using distilled water (DW), tap water (TP), and wastewater effluent (TW). Tap water was collected from the municipal system in Lublin, Poland, and the water was characterized by low NO_3_^−^ content (<2 mg/L), presence of chlorides (37 mg/L), Fe (52 mg/L), and pH = 7.2. The TWW was collected from the mechanical-biological municipal wastewater treatment plant in Lublin “Hajdów”. The TWW was characterized by low biological (5.7 mg/L) and chemical (35.1 mg/L) oxygen demand, total suspended solids (up to 6.4 mg/L), and total nitrogen (10.72 mg/L) and phosphorous (0.27 mg/L) contents. In TW the presence of chlorides (35 mg/L), nitrates (<2 µg/L), and sulfates (41 mg/L) was determined. Kinetics was optimized in 5, 10, 30, 60, and 120 min and 24, 48 and 72 h. For the isotherm modeling, the time was set at six days. All of the samples were mixed (120 rpm) in the dark at room temperature. Then, the obtained solution was filtered (0.45 µm) and analyzed. The collected aliquots were analyzed by using an Agilent LC-DAD chromatograph (1260 Infinity II) equipped with an InfinityLab Poroshell 120 EC-C18 column (3.0 × 150 mm, 2.7 µm), using the mixture H_2_O + CH_3_COONH_4_ (pH = 6): ACN (*v*/*v* = 60:40) as mobile phase (flow 0.5 mL/min, temperature 30 °C), and analyzed at λ_NTZ_ = 240 nm, *R*_T_ = 3.99 min (the fitting of the calibration curve R^2^ = 0.9998).

The amount of adsorbed NTZ (c_s_, mg/g) was calculated considering the difference between the initial concentration of NTZ and equilibrium concentration (c_e_, mg/L) and volume of the solution (50 mL) and adsorbent mass (100 ± 2 mg). For kinetic and isotherm modeling the well-known models: pseudo-first-order (PFO), pseudo-second-order (PSO), Elovich (E), Intraparticle Diffusion (IPD), and Langmuir (L), Freundlich (F), Temkin (T), and Dubinin-Radushkevich (DR), respectively, were applied [52,53], and the respective constants and parameters were calculated from the linear forms of the equations. All experiments were run in duplicate and the mean values were presented. In the figures displayed, the error bars were added.

## 4. Conclusions

The application of pelletized biochar may be an effective solution for the pollution of water by different drugs. The process of sorption is efficient and enables one to remove NTZ in one hour. Although the obtained material differed in physicochemical properties, biochar was characterized by low surface area (with the highest value obtained for BCR—26.05 m^2^/g). Biochar obtained from residues from biogas production possessed also the mostly hydrophilic/polar surface (connected with the highest amount of O%). The surface of biochar was rich in functional groups (–OH, C=C, C=O, C–N, C=N, N–H) whereas the surface of BCR was graphitized. The presence of voids and channels in the structure of BC was evidenced in SEM images. The highest amount of adsorbed NTZ (almost 14 mg/g) was observed on BCR. In general, the process of sorption was rather fast and the sorption equilibrium was maintained for one hour indicating potential practical application. In general, the maximum adsorption capacity of all samples increased in the following order BCS < BCF < BCR. The process of NTZ adsorption followed mainly PSO kinetics indicating that the adsorption rate was combined with the number of binding sites and governed by chemisorption. The presence of inorganic ions had a detrimental effect on sorption and carbonates were the most effective in hindering the process. The environmentally relevant concentration of DOM (10 mg/L) did not affect the process (whereas, in excess of DOM, adsorption was significantly lower). The model studies were supported by the results with a real water matrix, in which only a 15% reduction of adsorption efficiency was noted in wastewater effluent. Depending on the applied feedstock, various models described NTZ adsorption onto tested biochars. For BCS, the Temkin model revealed the highest fitting, whereas Dubinin-Radushkevich for BCF and Langmuir for BCR. The process of the adsorption of NTZ onto tested samples was governed by different interactions including electrostatic, hydrophobic interactions, and hydrogen bonding between carbonyl groups (COO^−^) and hydroxyl oxygen or protonated amine. In summary, the application of carbonaceous adsorbents in pelletized form is effective and carbonaceous adsorbents can be easily modified to improve antiparasitic drug adsorption.

## Figures and Tables

**Table 1:** Antiparasitic drugs concentration [ng/L] in waters [10].

Compound	Groundwater	River Water	WWTP on River Doubs (Eastern France)	WWTP Effluents (Greece Volos)	WWTP Influents (Portugal Coimbra)	WWTP Effluents (Portugal Coimbra)	Hospital Effluents (Portugal Coimbra)
Metronidazole	4.9–35.6 Taiwan	0.05–13.51 Bangladesh	35.8	35.2	0–113	19.4–83.5	1559–12,315
Thiabendazole		(detection limit −1.6 China Jiulong River)			0–15.3	0.493–12.1	9.17–1746
Albendazole	1.9 Serbia				0–1.79		28.3

**Table 2:** The physicochemical properties of obtained BC.

	S_BET_ ^1^ (m^2^/g)	Vp ^2^ (cm^3^/g)	D ^3^ (nm)	Ash ^4^ (%)	N ^5^ (%)	C ^5^ (%)	H ^5^ (%)	O ^6^ (%)	H/C (-)	(O + N)/C (-)	O/C (-)	TC ^7^ (mg/g)	IC ^8^ (mg/g)	TOC ^9^ (mg/g)
BCS	0.093	0.001424	95.41	14.99	1.03	77.84	1.48	4.65	0.019	0.073	0.060	768.02	0.55	767.46
BCF	0.941	0.000798	14.04	6.70	0.96	86.71	1.67	3.96	0.019	0.057	0.046	878.52	0	878.52
BCR	26.05	0.004706	10.63	25.93	3.66	23.49	0.57	46.35	0.024	2.129	1.973	633.00	1.97	631.00

^1^ S_BET_—surface area (m^2^/g), ^2^ Vp—pore volume (cm^3^/g), ^3^ D—pore diameter (nm), ^4^ ash content after burning at 600 °C, ^5^ N, C, H content from CHN analysis (%), ^6^ O—content calculated considering ash and CHN content, ^7^ TC—total carbon, ^8^ IC—inorganic carbon, ^9^ TOC—total organic carbon.

**Table 3:** Kinetic parameters of adsorption of NTZ onto tested BC from the solutions 20 mg/L.

		PFO			PSO			Elovich			IPD		
	q_max_	k_1_	q_1_	R^2^	k_2_	q_2_	R^2^	α	β	R^2^	K_IPD_	β	R^2^
BCS	9.94	3.852	0.974	0.5269	0.0156	9.53	0.9914	1055.2	1.667	0.7665	0.05	6.21	0.5876
BCF	10.25	3.828	1.052	0.3022	0.0323	11.41	0.9922	147.0	1.139	0.6889	0.07	7.17	0.4461
BCR	13.44	13.996	1.066	0.9601	0.2371	13.80	0.9999	1720.6	0.985	0.7871	0.07	10.05	0.5205

q_exp_ (mg/L); k_1_ (min^−1^); q_1_, q_2_ (mg/L); R^2^ (-); k_2_ (g/mg min); α (mg/g min); β (g/mg); K_IPD_ (mg/g min^1/2^).

**Table 4:** Isotherm modeling of the obtained results.

	L				F			T			DR			
	Q_L_	K_L_ ×10^−3^	R^2^	R_L_	K_F_	n	R^2^	Q_T_	B	R^2^	Q_D_	E	B	R^2^
BCS	133.62	3.73	0.9762	0.67	0.653	0.961	0.9576	0.1272	144.53	0.9961	35.485	118.11	220.03	0.9461
BCF	34.93	12.17	0.8652	0.70	0.594	1.053	0.8432	0.1405	126.32	0.9838	43.898	110.08	253.27	0.9925
BCR	418.55	1.18	0.9996	0.67	0.464	1.033	0.9975	0.1152	143.77	0.9524	30.480	125.09	196.15	0.8294

Q_F_, Q_L_, Q_D_—(mg/g), K_L_—(L/g), B—(kJ/mol), E—(kJ/mol).

**Table 5:** Langmuir sorption capacities of various types of adsorbents for removal of metronidazole [10].

Material	pH	Temp. [°C]	Surface Area [m^2^/g]	Langmuir Sorption Capacity [mg/g]	Recovery/ Regeneration
Carbon materials F400	7	25	919	248.6	Yes
Carbon materials ACF	7	25	1441	249.2	Yes
Carbon materials MWCNT	7	25	144	49.8	Yes
Carbon materials CMK-3	7	25	917	219.3	Yes
Carbon materials MWCNT-HNO_3_	7	25	31	54.4	Yes
Carbon materials F400-HNO_3_	7	25	823	234.4	Yes
Commercial activated carbon (S)	6–7	25	1225	328.61	No
Commercial activated carbon (M)	6–7	25	1301	213.94	No
Petroleum coke activated carbon ©	6–7	25	848	287.53	No
Siris Seedpod activated carbon	7	30	1676.61	180.77	No
Siris Seedpod KOH activated carbon	7	30	1824.88	191.68	No
BCR—this study	6–7	25	26.05	418.55	-

## Data Availability

Data will be shared on the request.

## Sample Availability

Samples of the compounds are available from the authors.
